# Sequestration of PRMT1 and Nd1-L mRNA into ALS-linked FUS mutant R521C-positive aggregates contributes to neurite degeneration upon oxidative stress

**DOI:** 10.1038/srep40474

**Published:** 2017-01-17

**Authors:** Mi-Hee Jun, Hyun-Hee Ryu, Yong-Woo Jun, Tongtong Liu, Yan Li, Chae-Seok Lim, Yong-Seok Lee, Bong-Kiun Kaang, Deok-Jin Jang, Jin-A Lee

**Affiliations:** 1 Department of Biotechnology, College of Life Science and Nanotechnology, Hannam University, Daejeon 34053, South Korea; 2 Department of Physiology, Seoul National University College of Medicine, Seoul 03080, South Korea; 3 Department of Life Science, Chung-Ang University, Seoul, 06974, South Korea; 4 Department of Applied Biology, College of Ecology and Environmental Science, Kyungpook National University, Sangju 37224, South Korea; 5 State Key Laboratory of Brain and Cognitive Science, Institute of Biophysics, Chinese Academy of Sciences, Beijing, China; 6 Department of Biological Sciences, College of Natural Sciences, Seoul National University, Seoul 08826, South Korea

## Abstract

Mutations in fused in sarcoma (FUS), a DNA/RNA binding protein, are associated with familial amyotrophic lateral sclerosis (ALS). However, little is known about how ALS-causing mutations alter protein-protein and protein-RNA complexes and contribute to neurodegeneration. In this study, we identified protein arginine methyltransferase 1 (PRMT1) as a protein that more avidly associates with ALS-linked FUS-R521C than with FUS-WT (wild type) or FUS-P525L using co-immunoprecipitation and LC-MS analysis. Abnormal association between FUS-R521C and PRMT1 requires RNA, but not methyltransferase activity. PRMT1 was sequestered into cytosolic FUS-R521C-positive stress granule aggregates. Overexpression of PRMT1 rescued neurite degeneration caused by FUS-R521C upon oxidative stress, while loss of PRMT1 further accumulated FUS-positive aggregates and enhanced neurite degeneration. Furthermore, the mRNA of Nd1-L, an actin-stabilizing protein, was sequestered into the FUS-R521C/PRMT1 complex. Nd1-L overexpression rescued neurite shortening caused by FUS-R521C upon oxidative stress, while loss of Nd1-L further exacerbated neurite shortening. Altogether, these data suggest that the abnormal stable complex of FUS-R521C/PRMT1/Nd1-L mRNA could contribute to neurodegeneration upon oxidative stress. Overall, our study provides a novel pathogenic mechanism of the FUS mutation associated with abnormal protein-RNA complexes upon oxidative stress in ALS and provides insight into possible therapeutic targets for this pathology.

Amyotrophic lateral sclerosis (ALS) is the most common motor neuron disease affecting upper and lower motor neurons in the brain and spinal cord^1^. The symptoms are progressive muscle weakness, atrophy, and spasticity, and patients typically die within 1–5 years after disease onset^2^. Although 80–90% of ALS cases are sporadic, about 10% are familial cases in which the disease has genetic components. After superoxide dismutase1 (SOD1) was identified as the first causative gene in familial ALS, more than 100 genes linked to ALS have been reported. Among these, mutations in core genes such as Chromosome 9 open-reading frame 72 (*C9orf72*), TAR DNA-binding protein (*TARDBP*), fused in sarcoma (*FUS*), valosin-containing protein (*VCP*), sequestosome 1 (*SQSTM1*, also known as *p62*), ubiquilin 2 (*UBQLN2*), and coiled-coil-helix-coiled-coil-helix domain containing 10 (*CHCHD10*), also occur in frontotemporal dementia (FTD), which is the second most common presenile dementia, suggesting that there is a link connecting these two different disorders^3^.

FUS is a DNA/RNA binding protein that was first identified as a fusion oncogene in human liposarcoma^4^. FUS is involved in many cellular processes such as transcriptional regulation, RNA splicing, DNA repair, genomic integrity, and responses to DNA damage^5^. In polarized neurons, FUS has been shown to regulate spine formation and maintenance of spine stability, transport of mRNA (such as actin-regulating mRNA) in dendrites, axonal transport of survival motor neuron (SMN) protein, regulation of stability of GluA1 (a subunit of AMPA subtype of ligand-gated glutamate receptors) mRNA, and activity-dependent synaptic homeostasis, all suggesting an important role in synaptic formation and function^6^,^7^,^8^,^9^.

Genetic mutations in FUS and their link to ALS were first reported in 2009^10^,^11^. In recent years, other rare genetic mutations within the *FUS* gene or FUS-positive inclusions have been discovered in patients with FTD, sporadic ALS, or essential tremor^5^. Mutations in the *FUS* gene account for 5% of familial ALS and less than 1% of FTD. Interestingly, most mutations in FUS are located in the end portion of the C-terminal region identified to be a proline-tyrosine nuclear localization signal, indicating that cytosolic mislocalization of FUS may contribute to neurodegeneration^12^.

Moreover, stress granule (SG) marker proteins are localized to large cytoplasmic FUS-positive inclusions in neurons and glial cells of diseased brain tissue, suggesting a pathogenic role of dysregulated SGs in neurodegeneration. SGs composed of translationally stalled mRNAs and several RNA-binding proteins are transient cytoplasmic foci (RNA granules) that appear under stress conditions^13^. Indeed, accumulating evidence shows that ALS-linked FUS mutants affect the dynamics of SGs, leading to abnormal cytoplasmic inclusions in primary neurons and in induced pluripotent stem cell-derived neurons, thereby indicating that they play a role in disease progression^14^,^15^. Furthermore, arginine methylation by protein arginine N-methyltransferase 1 (PRMT1) has been reported to regulate cellular localization of FUS, stress granule formation, and cellular toxicity of ALS-linked FUS mutants, indicating that post-translational modifications of FUS by PRMT1 affect its cellular function^16^,^17^,^18^,^19^.

It is critical to continue to investigate how specific ALS-linked mutations alter physiological protein-protein interactions or protein-RNA complex formation and cause cytosolic mislocalization of FUS and how abnormal protein-RNA complexes contribute to the cellular pathogenesis of ALS.

Although several studies have reported possible cellular pathogenic mechanisms associated with ALS-linked mutants, the exact mechanisms of specific FUS mutations remain unclear. ALS-causing mutants show nuclear or cytoplasmic aggregates, which sequester nuclear or cytosolic proteins or RNAs. Dysregulated protein-protein or protein-RNA interactions caused by ALS-linked mutants might impair RNA metabolism and RNA transport. This in turn can affect physiological neuronal morphology and function by sequestering several proteins and RNAs, which likely contributes to neurodegeneration. Moreover, what regulates this sequestration or association of proteins and RNA into ALS-linked aggregates is largely unknown.

In our study, to identify proteins with differential affinities to ALS-linked mutants than to FUS-WT (wild type), we performed co-immunoprecipitation (co-IP) of FLAG-tagged FUS-WT, FUS-R521C, and FUS-P525L, and liquid chromatography-mass spectrometry (LC-MS). We found that PRMT1 was avidly associated with FUS-R521C but not with FUS-WT or FUS-P525L. Although PRMT1 activity is not required for their strong association, RNAs and RGG2-ZnF-RGG3 domain of FUS mediate the formation of FUS-R521C/PRMT1 complexes. The loss of PRMT1 reduced total neurite length, while its expression partially rescued neurite morphology changes caused by FUS-R521C, indicating its effects on neurite regulation. We also found that the mRNA of Nd1-L, an actin-stabilizing protein that might be responsible for neurite phenotype, was sequestered into the stable FUS/PRMT1 complex. Indeed, expression of Nd1-L partially rescued neurite length, whereas reduction of Nd1-L mRNA further reduced neurite length in FUS-R521C-expressing neurons. These results suggest a novel cellular pathogenic mechanism in which the FUS-R521C/PRMT1/Nd1-L mRNA complex acts on neurite degeneration associated with ALS upon oxidative stress.

## Results

### FUS-R521C associates more avidly with PRMT1 than with FUS-WT or FUS-P525L

**Figure 1:**
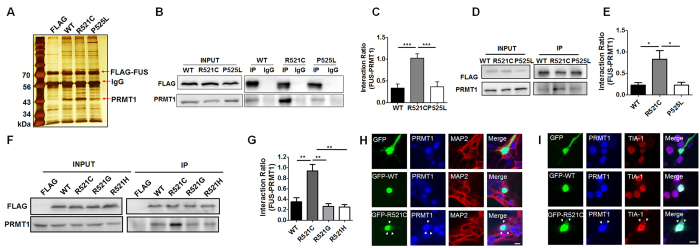
PRMT1 was more associated with ALS-associated FUS-R521C than with FUS-WT or FUS-P525L. (**A**) Silver-stained SDS-PAGE gels of the immunoprecipitates from HEK293T cell lysates expressing FLAG-vector (FLAG), FLAG-FUS-WT (WT), FLAG-FUS-R521C (R521C), and FLAG-FUS-P525L (P525L). (**B**) Immunoprecipitation (IP) and western blot analysis using HEK293T cell lysates expressing FUS-WT (WT), FUS-R521C (R521C), or FUS-P525L (P525L) with anti-FLAG (or IgG) and anti-PRMT1 antibodies. Extended blot images including these data are presented in Supplementary Fig. 5A. (**C**) Bar graph showing the ratios of association of PRMT1 and FUS. The data are from independent experiments and presented as mean ± SEM (n = 6). ***p < 0.001, one-way analysis of variance (ANOVA) followed by Tukey’s multiple-comparison test. (**D**) Immunoprecipitation and western blot analysis using anti-FLAG and anti-PRMT1 antibodies in neuronal lysates infected with AAV-FLAG-FUS-WT (WT), AAV-FLAG-FUS-R521C (R521C), or AAV-FLAG-FUS-P525L (P525L) for 4–6 days. Extended blot images including these data are presented in Supplementary Fig. 5B. (**E**) Bar graph showing the ratios of association of PRMT1 and FUS in cortical neurons. The data are from independent experiments and presented as mean ± SEM (n = 3). *p < 0.05, one-way ANOVA followed by Tukey’s multiple-comparison test. (**F**) Immunoprecipitation and western blot analysis using anti-FLAG and anti-PRMT1 antibodies in HEK293T cell lysates expressing FLAG-vector (FLAG), FLAG-FUS-WT (WT), FLAG-FUS-R521C (R521C), FLAG-FUS-R521G (R521G) or FLAG-FUS-R521H (R521H). Extended blot images including these data are presented in Supplementary Fig. 5C. (**G**) Bar graph showing the ratios of association of PRMT1 and FUS in HEK293T cells. The data are from independent experiments and presented as mean ± SEM (n = 3). **p < 0.001, one-way ANOVA followed by Tukey’s multiple-comparison test. (**H**) Confocal images showing the cellular localization of FUS and PRMT1 in MAP2 (a neuronal marker)-positive neurons expressing GFP, GFP-FUS-WT (GFP-WT), or GFP-FUS-R521C (GFP-R521C). (**I**) Confocal images showing the cellular localization of FUS, PRMT1, and TIA-1 (a stress granule marker) in neurons expressing GFP, GFP-FUS-WT (GFP-WT), GFP-FUS-R521C (GFP-R521C). Transfected neurons were stained with anti-PRMT1, anti-MAP2, or anti-TIA-1 antibody. Arrowheads indicate FUS-PRMT1 cytosolic aggregates (**H**) and FUS/PRMT1-positive stress granule aggregates (**I**). Scale bar, 10 μm.

To identify the proteins abnormally associated with ALS-causing FUS mutants, FLAG-FUS-R521C, FLAG-FUS-P525L, or FLAG-FUS-WT was expressed in HEK293T cells^20^. Co-IP of cell lysates using FLAG antibodies was performed 48 h after transfection, and the proteins analyzed on silver-stained polyacrylamide gels from extracts of cells overexpressing each FLAG-tagged FUS protein. Silver-stained SDS-PAGE analysis of elutes showed a 43-kDa protein that was prominent in FUS-R521C samples, but weak in FUS-WT and FUS-P525L samples (Fig. 1A). LC-MS analysis revealed that the most abundant peptides in tryptic digests of the corresponding band were derived from the human protein arginine N-methyltransferase 1 (PRMT1), the predominant type-I PRMT in mammalian cells which is involved in gene transcription, DNA repair, signal transduction, and protein translocation^21^ (Supplementary Fig. 1). Our LC-MS findings further supports previous reports that FUS binds to PRMT1 in mammalian cell lines and neuronal cells *in vitro* and *in vivo*^16^,^17^,^18^,^22^,^23^.

To confirm the association between FUS and PRMT1 revealed by the LC-MS data, we conducted co-IP and western blot using anti-FLAG and anti-PRMT1 antibodies. Endogenous PRMT1 was pulled down with FLAG-FUS-WT, FLAG-FUS-R521C, or FLAG-FUS-P525L. Consistent with the silver staining results, the R521C point mutation in FUS specifically increased its interaction with PRMT1 relative to the WT or P525L mutation (Fig. 1B,C), raising the possibility that the association of FUS and PRMT1 could be altered by the specific ALS-linked mutation, R521C. We further confirmed that the R521C point mutation significantly enhanced the association between FUS and PRMT1 compared to WT or P525L mutant using cultured neuronal cell lysates infected with adeno-associated virus (AAV) expressing FLAG-FUS-WT, FLAG-FUS-R521C, or FLAG-FUS-P525L (Fig. 1D,E). Moreover, we found that the specific substitution of arginine for cysteine at position 521 in FUS (R521C) significantly enhanced its association with PRMT1, while this effect was not observed in other ALS-linked FUS mutants (R521H or R521G) (Fig. 1F,G).

Since the R521C point mutation in FUS specifically increased its interaction with PRMT1, we next examined the cellular localization of FUS-WT or FUS-R521C with PRMT1 in cultured cortical neurons expressing GFP, GFP-fused FUS-WT, or GFP-fused FUS-R521C. As expected, GFP-FUS-WT, GFP-FUS-R521C, or endogenous PRMT1 was primarily localized to the nucleus in MAP2-positive cortical neurons (Fig. 1H). However, ALS-linked FUS-R521C and endogenous PRMT1 were co-localized to the cytosol and formed cytosolic aggregates which are TIA-1-positive stress granules without any stress inducer (Fig. 1I). This finding clearly shows that the R521C point mutation in FUS causes cytosolic sequestration of PRMT1 into stress granules in cultured cortical neurons.

### PRMT1 was sequestered into methylated FUS-R521C-positive stress granule compared to FUS-WT

**Figure 2:**
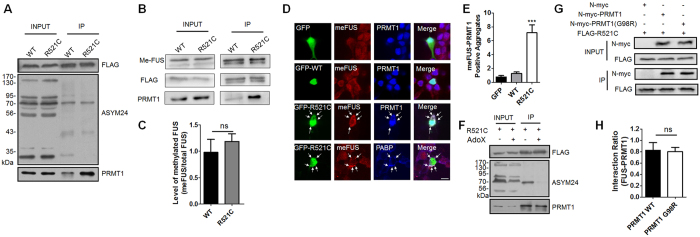
PRMT1 was sequestered into methylated FUS-R521C-positive aggregates. (**A**) Immunoprecipitation (IP) and western blot analysis using anti-FLAG, anti-PRMT1, or anti-ASYM24 antibody in HEK293T cell lysates expressing FLAG-FUS-WT (WT) or FLAG-FUS-R521C (R521C) in HEK293T cells. ASYM24 recognizes asymmetric dimethyl arginine. Extended blot images including this data are presented in Supplementary Fig. 6A. (**B**) IP and western blot analysis using anti-FLAG, anti-PRMT1, or anti-methylated FUS (me-FUS) antibody in HEK293T cell lysates expressing FLAG-FUS-WT (WT) or FLAG-FUS-R521C (R521C). Extended blot images including these data are presented in Supplementary Fig. 6B. (**C**) Bar graph showing the level of me-FUS normalized to immunoprecipitated total FUS. The data are from independent experiments and presented as mean ± SEM (n = 6). ns: not significant, Student’s *t*–test (two-tailed unpaired t-test, p = 0.32). (**D**) Confocal images showing cellular localization of FUS, me-FUS, and PRMT1 in neurons expressing GFP, GFP-FUS-WT (GFP-WT), or GFP-FUS-R521C (GFP-R521C). The arrow indicates me-FUS/PRMT1-positive cytosolic aggregates. Scale bar, 20 μm. (**E**) Bar graph showing the number of cytosolic aggregates of me-FUS and PRMT1 in neurons expressing GFP (n = 19), GFP-FUS-WT (WT, n = 19), or GFP-FUS-R521C (R521C, n = 19). The data are from independent experiments and presented as mean ± SEM (n = 3). ***p > 0.001, one-way ANOVA followed by Tukey’s multiple-comparison test. (**F**) Co-IP in the presence and absence of Adox, a methyltransferase inhibitor. HEK293T cells were pretreated with 25 μM Adox for 30 min prior to transfection. IP and western blot analysis use anti-FLAG, anti-endogenous PRMT1, anti-ASYM24 antibodies in HEK293T cell lysates expressing FLAG-FUS-R521C (R521C). Extended blot images including these data are presented in Supplementary Fig. 6C. (**G**) FLAG-FUS-R521C (FLAG-R521C) was transfected together with either N-myc-vector, N-myc-PRMT1-WT, or N-myc-PRMT1-G98R (a catalytic inactive mutant of PRMT1) into HEK293T cells. IP and western blot analysis use anti-FLAG, anti-N-myc antibodies. Extended blot images including these data are presented in Supplementary Fig. 6D. (**H**) Bar graph showing the ratios of association of PRMT1 and FUS. The data are from independent experiments and presented as mean ± SEM (n = 3). ns: not significant, two-tailed unpaired t-test, p = 0.51.

Various studies have reported that PRMT1 methylates arginine residues of FUS^16^,^18^,^19^. To test whether enhanced association of FUS-R521C with PRMT1 affects this process, we examined the methylation level of FUS-WT and FUS-R521C in HEK293T cells expressing FLAG-FUS-WT, or FLAG-FUS-R521C by using co-IP with anti-FLAG antibodies and western blot analysis with anti-PRMT1, anti-FLAG, and anti-ASYM24 (which recognize di-methylated proteins) antibodies. As shown in Fig. 2A, no substantial difference was observed in the global methylation of proteins associated with FUS-R521C. Furthermore, the level of methylation of FUS-R521C was not significantly enhanced compared to FUS-WT (Fig. 2B,C).

We next examined the cellular localization of methylated FUS (me-FUS) and PRMT1 in neurons expressing GFP, GFP-FUS-WT, or GFP-FUS-R521C by immunocytochemistry using anti-methylated FUS antibody, anti-PRMT1, or anti-PABP. In neurons expressing GFP or GFP-FUS-WT, me-FUS was mostly diffused in the nucleus and cytosol. However, in neurons expressing GFP-FUS-R521C, me-FUS was colocalized to FUS-R521C/PRMT1-positive stress granules without any stress induction, indicating that PRMT1 is partially sequestered into these me-FUS-R521C-positive stress granules (Fig. 2D,E).

To further characterize the abnormal interaction between FUS and PRMT1, we determined whether it requested PRMT1 activity of methyltransferase. We performed co-IP in the presence or absence of Adenosine-2′,3′-dialdehyde (Adox), an inhibitor of arginine methylation^24^. Incubation with Adox for 30 mins prior to transfection did not affect the abnormal association between FUS-R521C and PRMT1, indicating that such association is independent of arginine methylation (Fig. 2F). To further confirm this result, we performed co-IP using HEK293T cell lysates expressing FLAG-FUS-R521C and either N-myc vector, N-myc-PRMT1-WT, or N-myc-PRMT1-G98R (the catalytically inactive mutant PRMT1^25^). As shown in Fig. 2G,H, there is no significant difference in the association of FUS-R521C and PRMT1 between PRMT1-WT and PRMT1-G98R, indicating that PRMT1 methyltransferase activity is not essential for the enhanced association of FUS-R521C and PRMT1.

### RNA and RGG2-ZnF-RGG3 (RGG-2-3) domains are required for stable association of FUS complex with PRMT1

**Figure 3:**
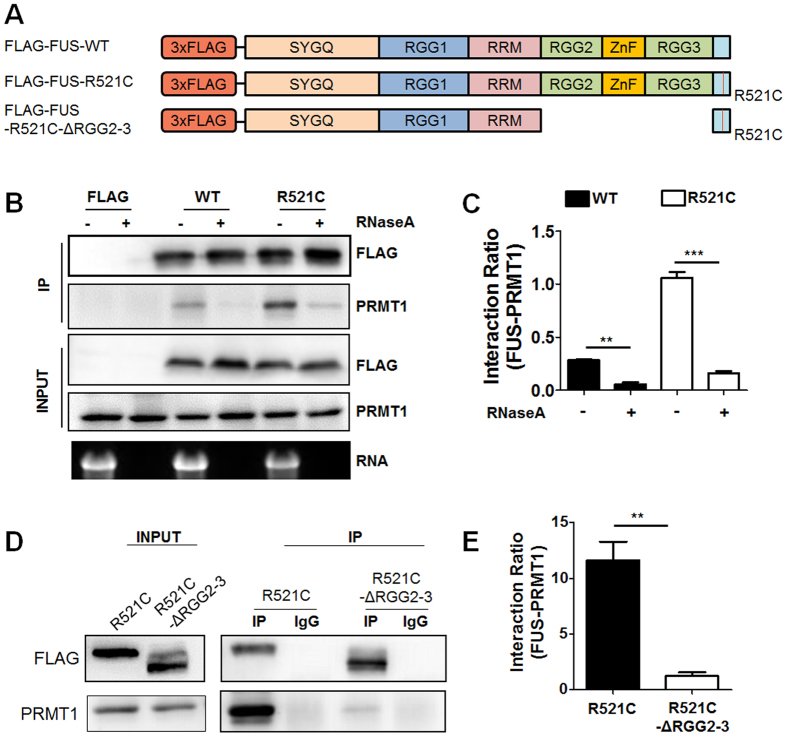
Association between FUS and endogenous PRMT1 is dependent on RNAs and RGG domains. (**A**) Schematic diagram of the domain structure of FUS-WT, FUS-R521C, and FUS-R521C deletion mutants with RGG2–ZnF-RGG3 (FUS-R521C-ΔRGG2-3). SYGQ (serine-tyrosine-glycine-glutamine-rich domain), RGG (arginine-glycine-glycine), ZnF (zinc finger), RRM (RNA recognition motif). (**B**) FLAG vector (FLAG), FLAG-FUS-WT (WT), or FLAG-FUS-R521C (R521C) was expressed in HEK293T cells. Forty-eight hours after transfection, immunoprecipitation (IP) was performed with anti-FLAG antibody using cell lysates expressing FLAG-vector, FLAG-FUS-WT or FLAG-FUS-R521C, with or without 200 μg/ml RNaseA, and western blot analysis was carried out with anti-FLAG and anti-PRMT1 antibodies. Samples with or without RNaseA were loaded in 1% agarose gel to check for RNA degradation. Extended blot or RNA gel images including these data are presented in Supplementary Fig. 7A. (**C**) Bar graph showing the ratios of association of PRMT1 and FUS in HEK293T cells expressing FLAG-vector (FLAG), FLAG-FUS-WT (WT) or FLAG-FUS-R521C (R521C) with or without RNaseA. The data are from independent experiments and presented as mean ± SEM (n = 3). **p < 0.01, ***p < 0.001, one-way ANOVA followed by Tukey’s multiple-comparison test. (**D**) FLAG-FUS-R521C (R521C) or FLAG-FUS-R521C-ΔRGG2-3 (R521C-ΔRGG2-3) were expressed in HEK 293 T cells. Cell lysates were used for immunoprecipitation (IP) with anti-FLAG (or IgG) antibodies. Western blot analysis was performed with anti-PRMT1 or anti-FLAG antibodies. Extended blot images including these data are presented in Supplementary Fig. 7B. (**E**) Bar graph showing the ratios of association of PRMT1 and FUS in HEK293T cells expressing FLAG-FUS-R521C (R521C) or FLAG-FUS-R521C-ΔRGG2-3 (R521C-ΔRGG2-3). The data are from independent experiments and presented as mean ± SEM (n = 3). **p < 0. 01, two-tailed unpaired t-test, p = 0.0038.

It has been reported that several RNA binding proteins, including FUS, require RNAs for formation of RNA granules^26^. The multi-step process of FUS aggregation has been shown to require RNA-dependent and RNA-independent processes^27^. Furthermore, it has also been reported that RNA seeds the higher-order assembly of FUS^28^. Therefore, we examined whether RNAs are required for stable FUS/PRMT1 complex formation. Cell lysates were incubated with RNase A for 2 h before co-IP with anti-FLAG antibodies was carried out. As shown in Fig. 3B,C, cell lysates treated with RNase A showed a complete loss of association of FUS-WT with PRMT1. Moreover, abnormal association of FUS-R521C with endogenous PRMT1 was significantly reduced, suggesting that RNAs are required for the association of FUS with PRMT1 (Fig. 3B,C).

To further confirm whether RNA binding and arginine methylation of FUS affect its association with PRMT1, we examined their association using co-IP with a deletion mutant of FUS RGG2-ZnF-RGG3 domains (FUS-R521C-ΔRGG2-3; Fig. 3A), which are involved in RNA recruitment and arginine methylation of RNA binding proteins^29^,^30^,^31^. As shown in Fig. 3D,E, association of PRMT1 with FUS-R521C-ΔRGG2-3, as compared to a full length FUS-R521C, was mostly reduced, suggesting that RGG2-ZnF-RGG3 domains of FUS-R521C are involved in the stable association of the FUS-R521C/PRMT1 complex. Therefore, these data suggest that RNAs and RGG2-ZnF-RGG3 domains are essential for the stable formation of FUS-R521C/PRMT1 complexes.

### Oxidative stress increased cytosolic FUS-R521C/PRMT1-positive SG aggregates which are regulated by PRMT1

**Figure 4:**
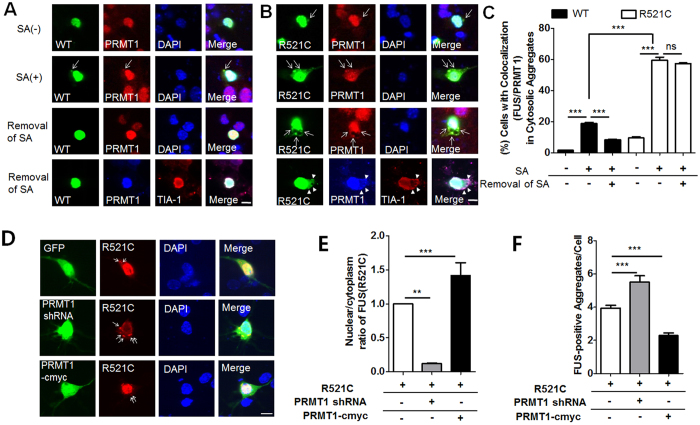
FUS-R521C-PRMT1 positive aggregates enhanced by oxidative stress remain after removal of stress and are regulated by manipulation of PRMT1. (**A**,**B**) Confocal images showing cellular localization of PRMT1 and either FUS-WT (**A**) or FUS-R521C (**B**) in neurons expressing GFP-FUS-WT (WT) or GFP-FUS-R521C (R521C) with or without oxidative stress [sodium arsenite (SA), 0.5 mM, 1 h] or 2 h after stress removal. Neurons were treated with SA (0.5 mM) for 1 h, 48 h after transfection. Arrow indicates FUS and PRMT1-positive aggregates. Arrowheads indicate FUS-R521C/PRMT1 positive stress granules. TIA-1: a stress granule marker. (**C**) Bar graph showing the percentage of cells with FUS and PRMT1 positive aggregates in neurons expressing FUS-WT (WT), FUS-R521C (R521C) with (or without) oxidative stress or 2 h after oxidative stress. The data are from independent culture experiments and presented as mean ± SEM (n = 3). For the quantification, we counted >200 neurons in each condition per experiment. ***p < 0.001, ns: not significant, one-way ANOVA followed by Tukey’s multiple-comparison test. (**D**) Representative confocal image of neurons expressing FUS-R521C(R521C) with or without PRMT1 by transfection of PRMT1-cmyc or pSUPER-GFP-PRMT1 shRNA (PRMT1 shRNA) under SA-induced oxidative stress. GFP: pSUPER-GFP-scramble. Scale bar, 20 μm. (**E**) Bar graph showing the nuclear-cytoplasmic ratio in neurons expressing either scrambled shRNA (n = 20), mouse PRMT1 shRNA (n = 24), or human PRMT1-myc (n = 20) in FUS-R521C-expressing neurons. The data are from independent culture experiments and presented as mean ± SEM (n = 3). **p < 0.01, ***p < 0.001 one-way ANOVA followed by Tukey’s multiple-comparison test. (**F**) Bar graph showing the number of FUS-positive aggregates in neurons expressing either scrambled shRNA (n = 34), mouse PRMT1 shRNA (n = 32), or human PRMT1-myc (n = 31). The data are from independent culture experiments and presented as mean ± SEM (n = 3). ***p < 0.001 one-way ANOVA followed by Tukey’s multiple-comparison test.

Previous studies including our own have shown cellular localization of ALS-linked FUS mutants into stress granules upon stress conditions^32^,^33^. Therefore, we examined cellular localization of PRMT1 and either FUS-WT or FUS-R521C upon oxidative stress and after the removal of oxidative stress. FLAG-FUS-WT, or FLAG-FUS-R521C was expressed in cultured cortical neurons and 48 h after transfection neurons were exposed to sodium arsenite (SA, 0.5 mM) for 1 h. As we expected, in the absence of oxidative stress PRMT1 was mostly colocalized to nuclear FUS-WT and partially localized to cytosolic FUS-R521C-positive aggregates (Fig. 4A,B). However, SA-induced oxidative stress significantly increased the number of cells with cytosolic FUS/PRMT1-positive aggregates in FLAG-FUS-R521C-expressing neurons than in FLAG-FUS-WT-expressing neurons, indicating that FUS-R521C together with PRMT1 preferentially localized to cytosolic stress granule upon oxidative stress (Fig. 4C).

Next, to examine their disassembly, cellular localization of either FUS-WT or FUS-R521C with PRMT1 was examined 2 h after the removal of SA. Interestingly, as shown in Fig. 4B,C, cytosolic FUS-R521C/PRMT1-positive SG aggregates were still present 2 h after removal of SA while cytosolic FUS-WT and PRMT1 disappeared, suggesting that their stable FUS-R521C/PRMT1 complex may contribute to stress-induced neurodegeneration during aging.

To investigate how PRMT1 affects the FUS-R521C/PRMT1 complex, we examined cytosolic FUS-positive aggregates upon oxidative stress in FUS-R521C-expressing neurons by overexpression or knockdown of endogenous PRMT1. We generated three different pSUPER-GFP-PRMT1 plasmids (#1–3) expressing shRNA against mouse PRMT1, examined their knockdown efficiency in mouse embryonic fibroblasts (MEFs), and used pSUPER-GFP-PRMT1 (#3) for further analysis (Supplementary Fig. 2A–C). We co-transfected pSUPER-GFP-PRMT1 #3 or pSUPER-GFP-scramble with FLAG-FUS-R521C into cultured cortical neurons. As shown in Fig. 4D–F, knockdown of PRMT1 increased cytosolic localization of FUS and FUS aggregates. Conversely, when we overexpressed PRMT1-cmyc together with FLAG-FUS-R521C, cytosolic FUS and aggregates were significantly reduced, suggesting that PRMT1 regulates the localization and aggregation of FUS-R521C (Fig. 4D–F).

### Manipulation of PRMT1 by overexpression or knockdown in FUS-R521C-expressing neurons regulates neurite morphology upon oxidative stress

**Figure 5:**
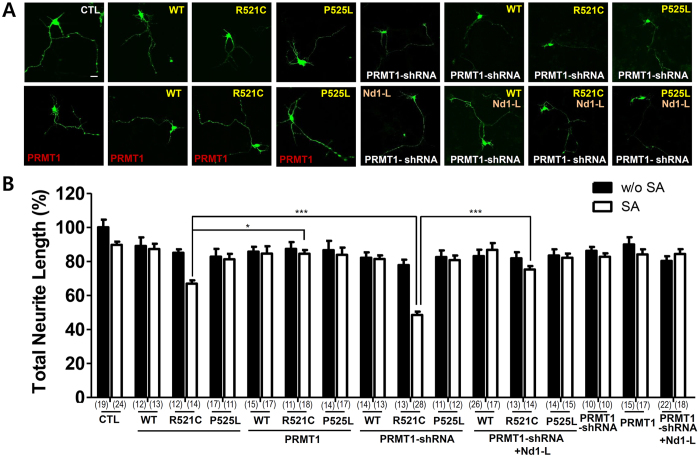
Overexpression or loss of PRMT1 regulates accumulation of cytosolic FUS aggregates and neurite morphology in cortical neurons upon oxidative stress. (**A**) Confocal image of neurite morphology of primary cultured neurons expressing pSUPER-GFP-scramble (CTL), FLAG-tagged FUS-WT (WT), FUS-R521C (R521C), FUS-P525L (P525L) with human PRMT1-cmyc (PRMT1), pSUPER-GFP-PRMT1 shRNA (PRMT1-shRNA), or pSUPER-GFP-scrambled shRNA. Neurons were treated with sodium arsenite (SA) (0.5 mM) for 1 h, 48 h after transfection. Scale bar, 20 μm. (**B**) Bar graph showing neurite length of neurons expressing pSUPER-GFP-scramble (CTL), FLAG-FUS-WT (WT), FLAG-FUS-R521C (R521C), or FLAG-FUS-P525L (P525L) together with either PRMT1-cmyc (PRMT1) or pSUPER-GFP-PRMT1 shRNA (PRMT1-shRNA). The data are from independent culture experiments and presented as mean ± SEM (n = 3). *p < 0.05, ***p < 0.001 one-way ANOVA followed by Tukey’s multiple-comparison test. The number (n) of neurons in each group is indicated in brackets.

Since the sequestration of PRMT1 by FUS-R521C affects the accumulation of cytosolic FUS-R521C aggregates, we investigated its contribution to neurite morphology. To determine the effects of FUS-R521C by manipulation of PRMT1 in cortical neurons, we examined neurite morphology in FUS-R521C-expressing neurons compared to GFP, FUS-WT, or FUS-P525L-expressing neurons with or without oxidative stress. As shown in Fig. 5, oxidative stress further reduced neurite length in FUS-R521C expressing neurons compared to GFP, FUS-WT, or FUS-P525L expressing neurons. Loss of PRMT1 by PRMT1 shRNA further enhanced neurite shortening in FUS-R521C-expressing neurons compared to the control (CTL: pSUPER-GFP-scramble) upon oxidative stress, while overexpression of PRMT1 significantly increased neurite length in FUS-R521C-expressing neurons compared to GFP, FUS-WT, or FUS-P525L expressing neurons (Fig. 5). Altogether, our results suggest that the sequestration of PRMT1 by FUS-R521C affects the accumulation of cytosolic FUS-R521C aggregates, which might contribute to neurite degeneration upon oxidative stress in cortical neurons.

### mRNA of Nd1-L, an actin-stabilizing protein, is sequestered into FUS-R521C-PRMT1 aggregates and its mRNA stability is affected by FUS-R521C

To investigate what types of RNA targets that might contribute to neurite degeneration are associated with the FUS/PRMT1 complex, we searched the literature for the known RNA targets of FUS in post-mitotic neurons^34^,^35^. Among several RNA targets of FUS^35^, Nd1-L mRNA has been shown to be one and to be transported along dendrites, suggesting that its dysregulation could affect neurite morphology and function^36^.

**Figure 6:**
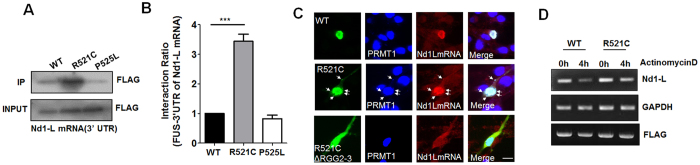
Nd1-L mRNA is sequestered into FUS-R521C-PRMT1 complexes. (**A**) Biotin-labeled 3′UTR RNA (7 μg) of Nd1-L was co-immunoprecipitated with FLAG-tagged FUS-WT (WT), FUS-R521C (R521C) or FUS-P525L (P525L) using streptavidin agarose beads and anti-FLAG antibody. Western blot analysis was performed using anti-FLAG antibodies. IP: immunoprecipitation. Extended blot images including these data are presented in Supplementary Fig. 8A. (**B**) Bar graph showing the ratios of association of FUS with 3′UTR of Nd1-L mRNA. Each ratio of association means the normalized FLAG-band intensity in HEK293T cells expressing FUS-WT (WT), FUS-R521C (R521C), FUS-P525L (P525L). The data are from independent experiments and presented as mean ± SEM (n = 3). ***p < 0.001 one-way ANOVA followed by Tukey’s multiple-comparison test. (**C**) *In situ* hybridization and immunocytochemistry in GFP-tagged FUS-WT (WT), FUS-R521C (R521C), or FUS-R521C-ΔRGG2-3 (R521C-ΔRGG2-3) expressing neurons using Cy3-conjugated probes (mRNA of Nd1-L, 200 nM) and anti-PRMT1 antibody. Arrows indicate cytosolic FUS-R521C-PRMT1-Nd1-L mRNA aggregates. Scale bar, 20 μm. (**D**) Nd1-L mRNA half-life was determined using actinomycin D treatment. FLAG-FUS-WT or FLAG-FUS-R521C was transfected into HEK293T cells. Forty-eighty hours after transfection, HEK293T cells expressing FUS-WT or FUS-R521C were treated with 5 μg/ml actinomycin D for 4 h. Samples for RNA analysis were collected immediately or at 4 h after actinomycin D treatment. Extended DNA gel images including these data are presented in Supplementary Fig. 8B.

To test whether Nd1-L mRNA is sequestered into FUS-R521C complexes, we performed mRNA-protein pull-down assay with biotinylated Nd1-L mRNA and streptavidin-agarose beads using HEK293T cell lysates expressing FLAG-FUS (WT, R521C, or P525L). As shown in Fig. 6A,B, the 3′-UTR of Nd1-L mRNA was more avidly associated with FUS-R521C than with FUS-WT or FUS-P525L.

To examine the cellular localization of FUS/PRMT1/Nd1L mRNA, we performed fluorescent *in situ* hybridization (FISH) in neurons expressing GFP-FUS-WT, GFP-FUS-R521C, or GFP-FUS-R521C-ΔRGG2-3, using fluorescence-labelled cDNA probes against Nd1-L mRNA and anti-PRMT1 antibody. Our FISH data indicated that endogenous Nd1-L mRNA and PRMT1 are colocalized to FUS-WT and FUS-R521C in the nucleus but not to FUS-R521C-ΔRGG2-3 which fails to associates with PRMT1 (Fig. 6C). Furthermore, our data showed colocalization of Nd1-L mRNA into FUS aggregates in the cell body and in the proximal region of neurons expressing FUS-R521C compared to neurons expressing FUS-WT, or FUS-R521C-ΔRGG2-3 (Fig. 6C), thus suggesting that Nd1-L mRNA is sequestered into FUS-positive aggregates and nucleus in neurons expressing FUS-R521C but not FUS-R521C-ΔRGG2-3. Furthermore, we found that overexpressed FUS-WT and FUS-R521C enhanced the fluorescence intensity of FISH probe against Nd1-L mRNA in the nucleus and FUS-positive aggregates (Fig. 6C), raising the possibility that the sequestration of Nd1-L by FUS-PRMT1 complex affects the stability of Nd1-L mRNA as well as its cellular localization.

To investigate whether the enhanced association of FUS-R521C with the 3′-UTR mRNA of Nd1-L indeed affects the stability of Nd1-L mRNA, we expressed FUS-WT or FUS-R521C in HEK293T cells and treated them with actinomycin D, which inhibits new transcription. To check the level of Nd1-L mRNA, we performed RT-PCR using cell lysates expressing FUS-WT or FUS-R521C and quantified the relative gene expression of Nd1-L mRNA immediately and at 4 h after actinomycin D treatment, 48 h after transfection. As shown in Fig. 6D, at 4 h after treatment, Nd1-L mRNA remained in cells expressing FUS-R521C, while its level was reduced in FUS-WT-expressing cells, suggesting that Nd1-L mRNAs might be trapped in stable FUS-RNA complexes. Therefore, these data suggest that Nd1-L mRNA is trapped and remained in FUS-R521C-complex in FUS-R521C-expressing cells compared to FUS-WT-expressing neurons.

### Expression of Nd1-L ameliorates neurite shortening caused by FUS-R521C upon oxidative stress, while loss of Nd1-L further exacerbates neurite shortening

Based on our co-IP experiments, Nd1-L mRNA was sequestered into FUS aggregates and remained trapped in FUS-R521C/PRMT1 complexes (Fig. 6). To elucidate whether this sequestration contributes to neurite shortening upon oxidative stress, we examined neurite morphology in FUS-R521C-expressing neurons compared to GFP, FUS-WT, or FUS-P525L expressing neurons while modulating Nd1-L.

**Figure 7:**
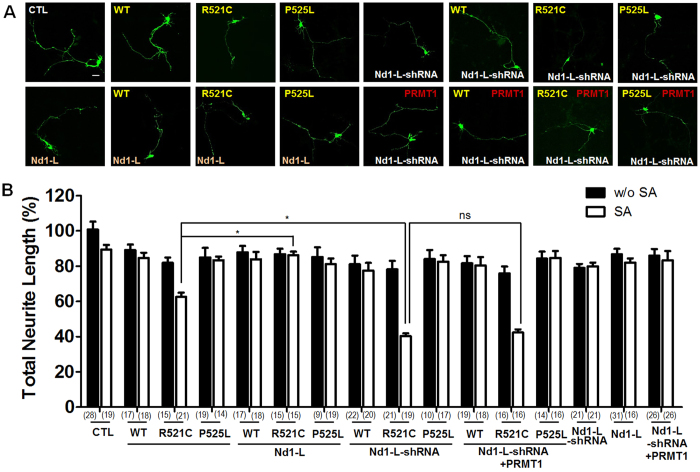
Expression of Nd1-L ameliorates neurodegeneration caused by FUS-R521C upon oxidative stress. (**A**) Confocal image of neurite morphology of neurons expressing pSUPER-GFP-scramble (CTL), FLAG-tagged FUS-WT, FUS-R521C, P525L by transfection of either Nd1-L-cmyc- or pSUPER-GFP-Nd1-L shRNA, pSUPER-GFP-scramble shRNA expressing vectors. Neurons were treated with sodium arsenite (0.5 mM, 1 h) 48 h after transfection. (**B**) Bar graph showing neurite length in pSUPER-GFP-scramble (CTL), FLAG-FUS-WT (WT), FLAG-FUS-R521C (R521C) or FLAG-FUS-P525L (P525L)-expressing neurons after Nd1-L-cmyc (Nd1-L) expression or pSUPER-GFP-Nd1-L shRNA (Nd1-L shRNA) expression with or without sodium arsenite (SA). The data are from independent culture experiments and presented as mean ± SEM (n = 3). Ns: not significant, *p < 0.05, one-way ANOVA followed by Tukey’s multiple-comparison test. The number (n) of neurons in each group is indicated in brackets.

We examined the effects of loss of Nd1-L on neurite shortening upon oxidative stress in neurons expressing FUS-R521C compared to the control (CTL: pSUPER-GFP-scramble) and to neurons expressing FUS-WT, or FUS-P525L with or without oxidative stress. We generated two different pSUPER-GFP-Nd1-L plasmids (#1–2) expressing shRNAs against mouse Nd1-L and checked its knockdown efficiency (Supplementary Fig. 3). We then transfected pSUPER-GFP-scramble, FLAG-FUS-WT, FLAG-FUS-R521C, or FLAG-FUS-P525L together with either pSUPER-GFP-Nd1-L (#2) or pSUPER-GFP-scramble into cultured cortical neurons. When Nd1-L expression was knocked down by transfection of pSUPER-GFP-Nd1-L (#2), neurite shortening caused by FUS-R521C was further aggravated, which was not the case in the control neurons, FUS-WT or FUS-P525L expressing neurons (Fig. 7). However, expression of Nd1-L-GFP partially rescued neurite shortening caused by FUS-R521C upon oxidative stress, indicating that actin stabilization by a pathophysiological level of Nd1-L might be important for the regulation of neurite length in cultured cortical neurons.

Furthermore, overexpression of PRMT1 did not rescue neurite shortening aggravated further by the loss of Nd1-L in FUS-R521C expressing neurons upon oxidative stress, while expression of Nd1-L partially but significantly rescued neurite length further reduced by loss of PRMT1 in FUS-R521C expressing neurons (Figs 5 and 7). These results suggest that the trap of Nd1-L mRNA into FUS-R521C-positive aggregates by the sequestration of PRMT1 can affect neurite shortening upon oxidative stress, leading to cellular pathogenesis associated with the FUS-R521C mutation.

## Discussion

Despite several recent studies on the pathogenic mechanisms of ALS-associated FUS mutants, little is known about how these different mutants affect neurite degeneration and phenotypic severities. Moreover, it is still largely unknown how these specific ALS-linked FUS mutations alter protein-protein and protein-RNA interaction, which could contribute to neurodegeneration upon stress.

In our study, we identified PRMT1 and the mRNA of Nd1-L as the components more avidly associated with ALS-linked FUS-R521C mutants than with FUS-WT or FUS-P525L. Although PRMT1 has been reported as a binding partner of FUS^16^,^18^,^23^,^26^,^37^, our study is the first to demonstrate that PRMT1 is associated with FUS-R521C more than with other FUS mutants. Based on our and other cellular studies, the FUS-R521C mutant is preferentially localized to SGs, leading to the formation of FUS-positive SG aggregates^38^,^39^,^40^. The rare FUS-P525L mutation leads to a more aggressive and rapidly progressive form of ALS in young patients compared to the FUS-R521C mutation, one of the most frequent mutations^30^,^41^. At the cellular level, FUS-P525L, due to the mutation in the proline-tyrosine nuclear localization signal (PY-NLS) domain, is mostly localized to the cytosol and forms cytosolic aggregates associated with SGs, while FUS-R521C is localized to both the nucleus and cytosolic SG aggregates^14^,^33^,^42^. This indicates that there is a specific cellular pathogenic mechanism associated with each mutation. Using LC-MS, we found that PRMT1 has higher affinity for the R521C mutant than WT or the P525L mutant, raising the possibility that its altered association with PRMT1 may be one of the major mechanisms for severe neurodegeneration.

PRMT1, which is responsible for almost 90% of cellular methylation by PRMTs in mammalian cells, is known to regulate RNA processing, transcriptional regulation, signal transduction, cellular localization, and DNA repair^21^. PRMT1 is localized in the nucleus and cytosol, and is highly mobile between these compartments^24^. Arginine methylation of FUS by PRMT1 has been known to affect its cellular localization, although the effect of this methylation on the localization of FUS depends on the cell type or the stress conditions^16^,^18^,^19^,^37^. In the present study, the stable complex of FUS-R521C with PRMT1 caused persistent sequestration of FUS/PRMT1 complex and accumulation of FUS-positive SG-aggregates, which lead to abnormal neurite morphology and might contribute to neurodegeneration, indicating that arginine methylation by PRMT1 regulates SG formation/assembly. A growing body of evidence also suggests that the impairment of SGs is linked to cellular pathogenesis of ALS associated with genetic mutations in C9ORF72, SOD1, TDP-43, Profilin-1^43^,^44^,^45^.

However, PRMT1 might also affect the activity of FUS as a transcriptional regulator via arginine methylation^22^. Accumulation of human mutant FUS-R495X in the cytoplasm caused nuclear depletion of PRMT1 *in vivo* in FUS-R495X transgenic mice, reducing methylation of its nuclear substrates^23^. Indeed, we also found that PRMT1 was more associated with FUS-R521C compared to FUS-WT in the nucleus and the cytosol (Supplementary Fig. 4). Therefore, it is plausible that sequestration of PRMT1 by ALS-linked FUS-R521C may cause PRMT1 loss-of-function mutations and impair its nuclear functions.

Furthermore, recent studies showed that the interaction of protein arginine methyltransferase 6 (PRMT6) with androgen receptor (AR) with poly-glutamine (Q) is significantly enhanced in an AR mutant associated with spinobulbar muscular atrophy (SBMA) leading to neurodegeneration^46^. Moreover, poly-Q expanded mutant huntingtin showed altered interaction with PRMT5, which resulted in reduction of PRMT5-mediated demethylation of histones^47^. Interestingly, FUS also acts as a modifier of a poly-Q expanded disease mouse model of Huntington’s Disease (HD)^48^. Therefore, it will be very interesting to investigate a molecular link between arginine methylation, FUS, cytosolic aggregation and stress granule assembly in motor neuron diseases.

The FUS-R521C mutation has indeed been reported to alter several protein-protein interactions. Neuronal aggregates formed by mutant FUS-R521C aberrantly sequestered survival motor neuron (SMN) protein through its enhanced association with SMN and concomitantly reduced SMN levels in axons, leading to axonal defects^7^. Moreover, mutant FUS proteins form stable complexes with FUS-WT and interfere with the normal interactions between FUS and histone deacetylase 1 (HDAC1) leading to DNA damage as well as profound dendritic and synaptic phenotypes^49^. Recently, it has been also reported that enhanced association between the FUS-R521C mutant and SMN reduced gems (gemini bodies) changed the steady state level of snRNA in transgenic mouse tissue and in human fibroblasts^50^.

Based on our co-IP study, specific substitution of arginine with cysteine (R - > C), but not histidine (R - > H) or glycine (R - > G), in FUS at the amino acid position 521 appears to be critical for the stable association of FUS-R521C with PRMT1. Cysteine residues are important for redox signaling^51^. The solubility of TDP-43 is regulated by direct stress-induced cysteine oxidation and disulfide bond formation^51^. Cysteine-generating ALS-linked TDP-43 mutants, such as G348C or S379C, produce abnormal disulfide cross-linking upon oxidative stress. In our study, the cysteine altered from arginine within FUS-R521C might have been affected by oxidation upon oxidative stress, which affects accumulation of SG aggregates of FUS-R521C. Therefore, it would be interesting to investigate the exact roles of disulfide species on the aggregation of FUS-R521C-positive stress granule.

A growing body of evidence has demonstrated the biochemical property of FUS as an RNA-binding protein that regulates RNA splicing^30^. Interestingly, RNA targets of FUS have long intron regions in their genes, indicating that FUS regulates alternative splicing or transcription. Although FUS RNA-binding sequences, such as GGUC/GUGGU, have been identified, recently accumulating evidence suggests that the FUS RNA-binding properties are not limited to specific target sequences, but rather depend on the secondary or tertiary structure of RNAs^52^,^53^,^54^. Indeed, it has been reported in an experiment using RNAs from the intron-exon boundary and 3′UTR of brain-derived neurotrophic factor (*Bdnf)* mRNA that the association between the low-complexity domain of FUS and RNAs is important for higher–order protein-RNA complexes^49^.

According to our co-IP data, stable FUS/PRMT1 complexes seem to be dependent on RNAs and RGG domains. FUS has been known to associate with many heterogeneous ribonuclear proteins, and their stable associations are also dependent on RNA. Consistent with these findings, RNA seeds the higher-order assembly of FUS^28^. ALS-linked FUS has been shown to form more stable FUS-RNA complexes in our and other studies^26^,^49^. Formation and accumulation of FUS-RNA-PRMT1 complexes could perturb several cellular pathways and cause abnormal neurite morphology. Abnormal axonal or dendritic phenotypes are common cellular phenotypes found in the brain tissue of patients with ALS and in ALS animal models^30^,^41^. It is still unknown, however, which types of RNA targets might contribute to neurite degeneration associated with ALS.

Among several potential RNA targets, we identified Nd1-L, a Kelch protein involved in the stabilization of actin filaments, as a potential component of FUS complexes^36^,^55^. Kelch repeats are known to be important for actin binding, protein folding, and protein-protein interaction^56^. FUS associates with the 3′UTR of Nd1-L, which lacks a GGUG-type motif and facilitates transport of Nd1-L mRNA intro dendritic spines upon mGluR5 (metabotropic glutamate receptor 5) activation^8^,^36^. Our protein-RNA co-IP and FISH data show that the FUS-R521C mutant can form stable complexes with Nd1-L mRNA and sequester it into FUS-R521C-positive aggregates. FUS can bind to Nd1-L mRNA, impairing its correct translation which leads to its sequestration into FUS aggregates. Indeed, overexpression of Nd1-L in FUS-R521C-expressing neurons rescued neurite shortening upon oxidative stress, suggesting that FUS/Nd1-L mRNA complexes contribute to abnormal neurite morphology. Nd1-L could therefore represent a molecular link between the regulation of actin cytoskeleton and neurite morphology^57^. Recent studies reported that mutations of profilin 1 (PFN1), an actin binding protein, affect cytoskeletal dynamics and aggregation of TDP-43^58^. Moreover, ALS/FTD-linked *C9ORF72* expansion has been shown to dysregulate actin dynamics in motor neurons^59^. Our study showed that Nd1-L expression rescued neurite shortening enhanced further by loss of PRMT1 in FUS-R521C expressing neurons, thus supporting that improper regulation of actin cytoskeleton might contribute to cellular pathogenesis associated with ALS. According to our data (Figs 5 and 7), overexpression of FUS (WT or R521C) itself does not significantly affect neurite shortening. When oxidative stress was exposed to neurons expressing FUS-R521C but not FUS-WT, neurite length was significantly reduced. Based on our results (Fig. 4B,C), oxidative stress enhanced accumulation of FUS-R521C-positive SGs which remained in the cytosol after removal of oxidative stress. Although a small portion of FUS-R521C-positive SGs and aggregates were accumulated in cytosol, oxidative stress-induced cytosolic aggregates of FUS-R521C/PRMT1/Nd1-L mRNA rather than the nuclear sequestration of Nd1-L mRNA into FUS-R521C/PRMT1 might impair cellular pathway involved in the regulation of neurite morphology. However, further studies are need to investigate how cytosolic aggregates of FUS-R521C/PRMT1/Nd1-L mRNA induced by oxidative stress exactly contribute to neurite degeneration.

Consistent with our data, recent evidence showed that FUS-R521C can form stable FUS-*Bdnf* mRNA or FUS-*MECP2* mRNA complexes, which impair transcription and/or RNA splicing suggesting that FUS mutants could disrupt target gene expression at a post-transcriptional level^49^,^60^. Recent RNA-seq transcriptome analyses have identified more target genes that are differentially regulated in FUS-WT and ALS-linked FUS mutants in cellular and animal models^49^.

One point of high impact in the present study is the identification of FUS/PRMT1/Nd1-L RNA complexes that can contribute to altered neurite morphology via the FUS-R521C mutation. This evidence opens to further research work to determine whether these abnormal complexes can interfere with RNA splicing in target genes, RNA transport granules, or local protein synthesis by synaptic activity. An additional topic for future studies is to investigate whether PRMT8, a neural-specific PRMT, is also sequestered from axonal and dendritic membranes into FUS-positive SG aggregates, and whether its alteration can cause synaptic deficits and neurodegeneration^18^.

## Methods

### Molecular cloning

Human cDNA of PRMT1-WT was obtained from Sino Biological Inc. (North Wales, PA). FUS-WT and FUS mutants including FUS-R521C, FUS-P525L, FUS-R521G, FUS-R521C-ΔRGG2-3, PRMT1-G98R, and Nd1-L were created by PCR using specific primers (Supplementary Table 1). The amplification products FUS-WT or FUS mutants were inserted into p3XFLAG-CMV7.1 (Sigma Aldrich, St. Louis, MO) and pEGFP vectors (Clontech, Mountain View, CA) using HindIII and BamHI enzymes restriction sites. The amplification products PRMT1-WT or PRMT-G98R were inserted into pCMV-Myc-N (Clontech, Mountain View, CA) using EcoRI and KpnI enzymes restriction sites. The amplification product Nd1-L was inserted into pcDNA™3.1/myc-His vectors (Thermo Fisher Scientific) using EcoRI and KpnI restriction sites.

### Cell cultures, transfection, viral infection, and immunocytochemistry

All experimental procedures were approved by the Institutional Animal Care and Use Committee of Hannam University and in accordance with their guidelines. Preparation of neuronal cultures from ICR mice brains, transfection and immunocytochemistry were conducted as previously described^61^. Plasmid DNA was transfected into primary cortical neuronal cells using Lipofactamine 2000 (Invitrogen, part of Thermo Fisher Scientific, Carlsbad, CA), at 3–5 days *in vitro* (DIV) according to manufacturer’s procedure.

To generate 3xFLAG-FUS fusion AAV constructs, we performed PCR using each primer sets (Supplementary Table 1). PCR products were inserted into the pAAV-CW3SL-GFP vector using Acc I and Xho I enzymes restriction sites. The titer of the virus production was quantified with real-time PCR. For the generation of the virus, 3xFLAG-FUS fusion AAV constructs and viral packaging vectors were transfected into HEK293T cells. The 3xFLAG-FUS fusion AAV virus was diluted in phosphate-buffered saline (PBS) and infected into primary neuronal cells at DIV 3 for 6 d. For immunocytochemistry, anti-FLAG (1:100; Sigma Aldrich, St. Louis, MO), anti-PRMT1 (1:100; Cell Signaling Technology, Danvers, MA), anti-FUS (1:100; Santa Cruz Biotechnology, Dallas, TX), anti-PABP (1:100; Abcam, Cambridge, UK), anti-TIA1 (1:100; Santa Cruz Biotechnology, Dallas, TX), anti-methylated FUS (1:100, Funakoshi Co., Tokyo, Japan), and anti-MAP2 (1:100 or 1:1000, Merck Millipore, Billerica, MA) were used as primary antibodies.

### Immunoprecipitation and Western blotting in HEK293T cells and cortical neurons

HEK293T cells or cultured cortical neurons expressing FLAG-FUS (WT, R521C, or P525L) were prepared in IP lysis buffer (50 mM Tris-HCl, pH 7.5, 150 mM NaCl, 2 mM EDTA, 1% NP40, and protease and phosphatase inhibitors). The cell lysates were incubated with anti-FLAG antibodies (F1804, Sigma Aldrich, St. Louis, MO) overnight at 4 °C. The following day, antibody-protein complex cell lysates were incubated with protein G-agarose beads for 7 h at 4 °C before being washed with IP lysis buffer two times at 4 °C. For western blot analysis we used Anti-FLAG (1:20000; Sigma Aldrich, St. Louis, MO), anti-GAPDH (1:10000; Merck Millipore, Billerica, MA), anti-methylated FUS (1:100, Funakoshi Co., Tokyo, Japan), anti-PRMT1 (1:1000; Cell Signaling Technology, Danvers, MA), anti-ASYM24 (1:1000, Merck Millipore, Billerica, MA), anti-Lamin A/C (1:800, Santa Cruz Biotechnology, Dallas, TX), and anti-N-Myc (1:1000, Clontech, Mountain View, CA) antibodies as primary antibodies, and anti-HRP rabbit, mouse, goat, or rat ones as secondary antibodies.

### Silver staining and LC-MS analysis

Silver staining was performed using a PageSilver™ Silver Staining Kit (#K0681; Thermo Fisher Scientific, Waltham, MA) according to the manufacturer’s instructions. Briefly, after SDS electrophoresis, the gel was placed in 50 ml fixing solution 1 [25 ml ethanol, 5 ml glacial acetic acid, diluted to 50 ml with deionized water (DW)] and 50 ml fixing solution 2 (30 ml ethanol diluted to 100 ml with DW) for 10 min at room temperature. After rinsing the gel three times with DW, 50 ml of sensitizing solution (0.2 ml sensitizer concentrate diluted to 50 ml with DW) was added, and the sample was shaken for 1 min. The gel was stained with staining solution containing 27 μl formaldehyde after washing. The sample was washed and treated with the developing solution. When the bands appeared, the developing solution was removed and the stop solution was added for 5 min. LC/MS analysis has been done by Yonsei Proteome Research Center.

### RNase treatment

After homogenization of the IP, we centrifuged the cell lysate at 4 °C and 13,000 rpm. The supernatant was transferred to a new 1.5 ml tube, after which the samples were treated with RNase (200 μg/ml) and incubated for 2 h at 4 °C. Following RNA elution, the RNA was loaded onto an agarose gel (2%) to confirm via electrophoresis that the RNase treatment had been successful.

### Generation of pSUPER-GFP-PRMT1, Nd1-L shRNA and their efficiency test in MEF cells

Sense and antisense RNA of each shRNA for mouse PRMT1, mouse Nd1-L, and scramble were annealed and inserted into pSuper.neo + GFP vector (OligoEngine, Seattle, WA) using HindIII and BglII enzymes restriction sites according to the manufacturer’s instructions. To test the knockdown efficiency of each mouse PRMT1 or Nd1-L shRNA, these shRNAs were reversely transfected into mouse embryonic fibroblasts (MEF) using Lipofectamine 2000 (Invitrogen, part of Thermo Fisher Scientific, Carlsbad, CA) according to the manufacturer’s instructions.

### Nd1-L mRNA stability assay

For the mRNA stability assay, FUS-WT and FUS-R521C were reverse transfected into HEK293T cells using Lipofectamine 2000 (Invitrogen, part of Life Technologies, Carlsbad, CA). At 48 h after the transfection, cells were treated with actinomycin D (5 μg/ml) and sodium arsenite (0.5 mM/ml) for 4 h. This treatment was not applied to the control cells. Isolation of total RNA was performed with TRIzol (Invitrogen, part of Thermo Fisher Scientific, Carlsbad, CA). cDNA was synthesized with DNase I-treated total RNA using the Superscript III First-Strand Synthesis System (Invitrogen, part of Thermo Fisher Scientific, Carlsbad, CA) according to the manufacturer’s instructions. Semi-quantitative PCR was carried out with cDNA using gene specific primers (Supplementary Table 1).

### Quantification of cellular localization, number of FUS-positive aggregates, total neurite length, and statistical analysis

Localization of proteins and mRNA under investigation in the cytoplasm or nucleus was measured using ImageJ software (U.S. National Institutes of Health, Bethesda, MD) as described previously^62^. Confocal images were loaded into ImageJ. Using plugins (ROI/Multi Measure), the background and at least three regions of interest were selected using the drawing tool. Intensity measurements were quantified as nuclear-cytoplasmic ratios. Anti-MAP2 or anti-TUJ1 positive neurites were used for the quantification of the neurite length as described previously^63^. Neuronal images were then obtained using an LSM 700 confocal laser scanning microscope (Carl Zeiss, Oberkochen, Germany). The neurite length was measured and quantified using the Image J (U.S. National Institutes of Health, Bethesda, MD), Photoshop CS3 (Adobe Systems, San Diego, CA), and Prism5 (GraphPad Software, La Jolla, CA) programs. The lengths of 10 to 30 neurons in each experimental group were measured and quantified from three to six independent experiments. The percentage of cells with FUS- and PRMT1-positive staining in cytosolic aggregates were counted. Student’s *t*-test (Two-tailed unpaired t-test) or one-way ANOVA in conjunction with Tukey’s multiple comparison test as a post-hoc analysis, were carried out for statistical analysis using Prism5 program (GraphPad Software, La Jolla, CA).

### mRNA-protein pull-down assay

We performed the mRNA-protein pull-down assay by modifying a method described previously^64^. Human Nd1-L 3′UTR was subcloned into a pGEM-T Easy vector (Promega, Madison, WI) and *in vitro* transcription of pGEM-human Nd1-L 3′UTR was performed using T7 RNA polymerase (Promega, Madison, WI) and the nucleotide analogs Bio-17-ATP and Bio-11-CTP (Enzo Life Sciences Inc., Farmingdale, NY). Biotinylated RNA was analyzed by agarose-gel electrophoresis and quantified using the NanoDrop spectrophotometer 2000 (Thermo Fisher Scientific, Wilmington, DE). Cell lysates of 3× FLAG fusion protein (FUS-WT, FUS-R521C, FUS-P525L)-transfected HEK293T cells were prepared using a lysis/binding buffer (50 mM Tris-HCl, pH 7.5, 150 mM NaCl, 1 mM EDTA, 5% glycerol, 0.1% Triton-X 100, 1.5 mM DTT, 0.2 mg/ml heparin, 0.2 mg/ml yeast tRNA, 0.25% BSA, 40 U/ml RNase inhibitor, and a protease inhibitor cocktail). Biotinylated RNA (7 μg) was mixed with pre-cleared cell lysate. Streptavidin-agarose bead (Thermo Fisher Scientific, Waltham, MA) (30 μl) was added to the cell lysate, and the mixture was further incubated at 4 °C for 4 h. Beads were washed five times with washing buffer containing 50 mM Tris-HCl (pH 7.6), 150 mM NaCl, 5% glycerol, 0.1% Triton X-100, 1 mM DTT and 40 U/ml RNasin (Promega, Madison, WI). RNA-protein binding was detected using anti-FLAG (F1804, Sigma Aldrich, St. Louis, MO).

### Fluorescence *in situ* hybridization (FISH)

Primary cultures of mouse cortical neurons on glass were fixed in 4% paraformaldehyde (PFA) at room temperature for 10 min. After washing with 1X PBS twice, cells were permeabilized with 0.1% Triton X-100 for 10 min, rinsed with 1X PBS, and treated with 1.5% BSA for 30 min before staining with primary antibodies (anti-MAP2, anti-PRMT1 and anti-FLAG). After incubation with primary antibodies, cells were incubated with secondary antibodies conjugated with Alexa 488 and DyLight 405 (Jackson Immuno Research Laboratories, West Grove, PA). Immuno-stained cells were rehydrated with rehydration buffer [2X saline sodium citrate (SSC) and 50% formaldehyde] at room temperature for 5 min. Cy3-conjugated probes (Supplementary Table1) were diluted to 500 nM in hybridization buffer (2X SSC, 25% formaldehyde, 10% dextran sulfate, and 0.005% BSA) and incubated at 37 °C for 4 h or overnight. The hybridized samples were rinsed in rehydration buffer at 37 °C for 30 min and mounted onto glass slides with Vectashield mounting medium (Vector Laboratories, Burlingame, CA).

## Additional Information

**How to cite this article**: Jun, M.-H. *et al*. Sequestration of PRMT1 and Nd1-L mRNA into ALS-linked FUS mutant R521C-positive aggregates contributes to neurite degeneration upon oxidative stress. *Sci. Rep.* **7**, 40474; doi: 10.1038/srep40474 (2017).

**Publisher's note:** Springer Nature remains neutral with regard to jurisdictional claims in published maps and institutional affiliations.

## Supplementary Material

Supplementary Information
