# Supplementary material for: Sequestration of PRMT1 and Nd1-L mRNA into ALS-linked FUS mutant R521C-positive aggregates contributes to neurite degeneration upon oxidative stress
Source: Sci Rep. 2017 Jan 17;7:40474. doi: 10.1038/srep40474 (PMC5240339; doi:10.1038/srep40474)
Supplement: Supplementary Information [file srep40474-s1.pdf]

Manuscript#: SREP-16-30071B

Sequestration of PRMT1 and Nd1-L mRNA into ALS-linked FUS mutant R521C-positive aggregates contributes to neurite degeneration upon oxidative stress

Mi-Hee Jun[1,7], Hyun-Hee Ryu[1,2,6,7], Yong-Woo Jun[3], Tongtong Liu[4], Yan Li[4], Chae-Seok Lim[5], Yong-Seok Lee[2], Bong-Kiun Kaang[5], Deok-Jin Jang[3*], and Jin-A Lee[1*]

[1]Department of Biotechnology, College of Life Science and Nanotechnology, Hannam University, Daejeon 34053, South Korea;[2] Department of Physiology, Seoul National University College of Medicine, Seoul 03080, South Korea; [3]Department of Applied Biology, College of Ecology and Environmental Science, Kyungpook National University, Sangju 37224, South Korea; [4]State Key Laboratory of Brain and Cognitive Science, Institute of Biophysics, Chinese Academy of Sciences, Beijing, China; [5]Department of Biological Sciences, College of Natural Sciences, Seoul National University, Seoul 08826, South Korea; [6]Department of Life Science, Chung-Ang University, Seoul, 06974, South Korea.

[7]These authors contributed equally to this study.

*Co-Correspondence to: Jin-A Lee, leeja@hnu.kr; Deok-Jin Jang, jangdj@knu.ac.kr

Protein	Mass	Score	Queries matched
Protein arginine N-methyltransferase1 variant 1 (Homo sapiens)	39602	912	109
Chain A, Human Serum Albumin Complexed With Myristate And Aspirin	66454	176	14
Arginine N-methyltransferase (Homo sapiens)	38835	145	14
unnamed protein product (Homo sapiens)	69295	130	12
B cell antibody heavy chain variable region (Homo sapiens)	13573	78	11
Ankyrin-3 isoform 1 (Homo sapiens)	480404	74	16
Glycoprotein receptor gp330/megalin precursor (Homo sapiens)	521976	73	12
Fibrillin 2 (congenital contractural arachnodactyly) variant (Homo sapiens)	215625	72	18
CENPE variant protein (Homo sapiens)		71	17
Fibrillin-2 precursor (Homo sapiens)	314773	71	21
Centromere-associated protein E (Homo sapiens)		71	18
Firbrillin-2 (Homo sapiens)	314346	70	22
Nebulin, isoform CRA_a (Homo sapiens)		70	26

Supplementary Figure 1. List of FUS-R521C interacting proteins from LC-MS analysis.

Identification of interacting proteins associated with FUS-R521C by LC-MS.

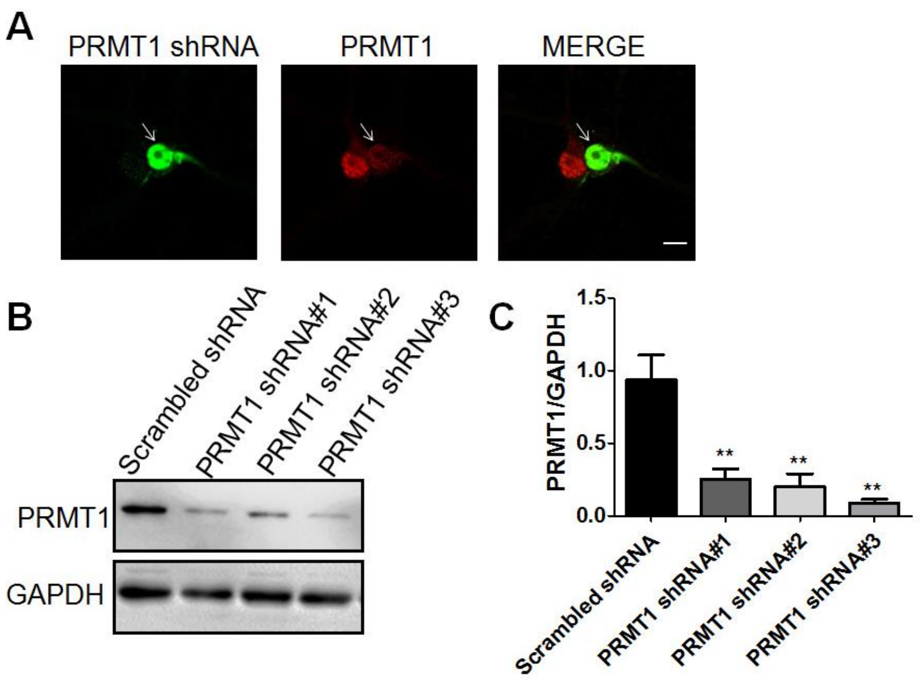

Supplementary Figure 2. Mouse PRMT1 is reduced by the expression of shRNA against PRMT1 in neurons.

(A) Confocal images showing that expression of mouse PRMT1 was reduced in PRMT1 shRNA (#3)-transfected mouse cortical neurons. Neurons were immunostained with anti-PRMT1 antibody 48 h after transfection. Green signal: GFP, Red signal: endogenous PRMT1. Scale bar: 20 μm.(B) Endogenous PRMT1 was decreased in mouse embryonic fibroblasts (MEFs) by three different mouse shRNA-expressing vectors [pSUPER-GFP-PRMT1 shRNA (#1-3)]. Western blot analysis was performed in MEF cells lysate expressing shRNA against mouse PRMT1 48 h after transfection with anti-PRMT1 and anti-GAPDH antibodies. (C) Bar graph showing PRMT1 level normalized with GAPDH in HEK293T cells expressing each shRNA. The data are from independent experiments and presented as mean ± SEM (n = 3). **, $p < 0.01$, one-way ANOVA followed by Tukey's multiple-comparison test.

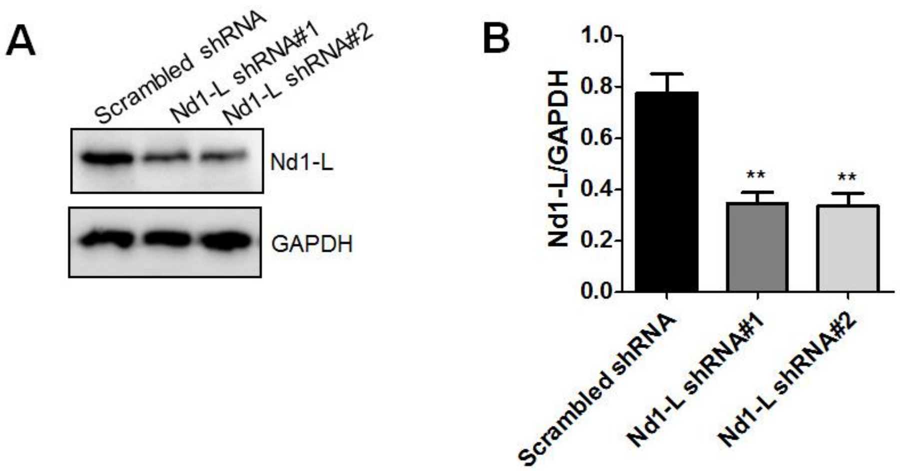

Supplementary Figure 3. Mouse Nd1-L is reduced by expression of shRNAs in MEFs.

Knockdown efficiency of mouse shRNA and human Nd1-L siRNA. (A) Two different Nd1-L shRNA-expressing vectors (pSUPER-GFP- Nd1-L shRNA) against Nd1-L were transfected into MEF cells. Endogenous Nd1-L was decreased in mouse embryonic fibroblasts (MEFs) by two different mouse shRNA-expressing vectors [pSUPER-GFP-Nd1-L shRNA (#1-2)]. Western blot analysis was performed in MEF cells lysates expressing shRNA against mouse Nd1-L 48 h after transfection using anti-Nd1-L and anti-GAPDH antibodies. (B) Bar graph showing Nd1-L level normalized to GAPDH in HEK293T cells expressing each shRNA. The data are from independent experiments and presented as mean ± SEM (n = 3). ***p* < 0.01, one-way ANOVA followed by Tukey's multiple-comparison test.

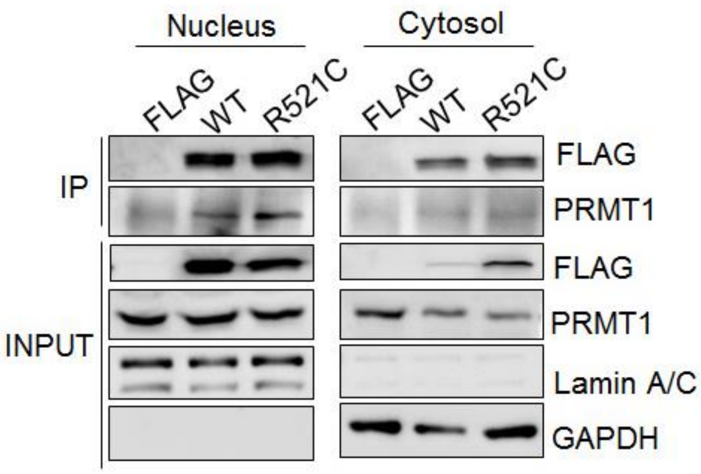

Supplementary Figure 4. PRMT1 is more associated with FUS-R521C compared to FUS-WT in the nucleus and cytosol.

FLAG-FUS-WT or FLAG-FUS-R521C was transfected in Hela cells. Forty-eight hours after transfection, immunoprecipitation was carried out using nuclear, or cytosolic cell lysate after fractionation with anti-FLAG antibodies, and Western blot was performed using anti-FLAG, anti-endogenous PRMT1, anti-LaminA/C, or anti-GAPDH antibodies. LaminA/C: a nuclear marker protein, GAPDH: a cytosolic marker protein.

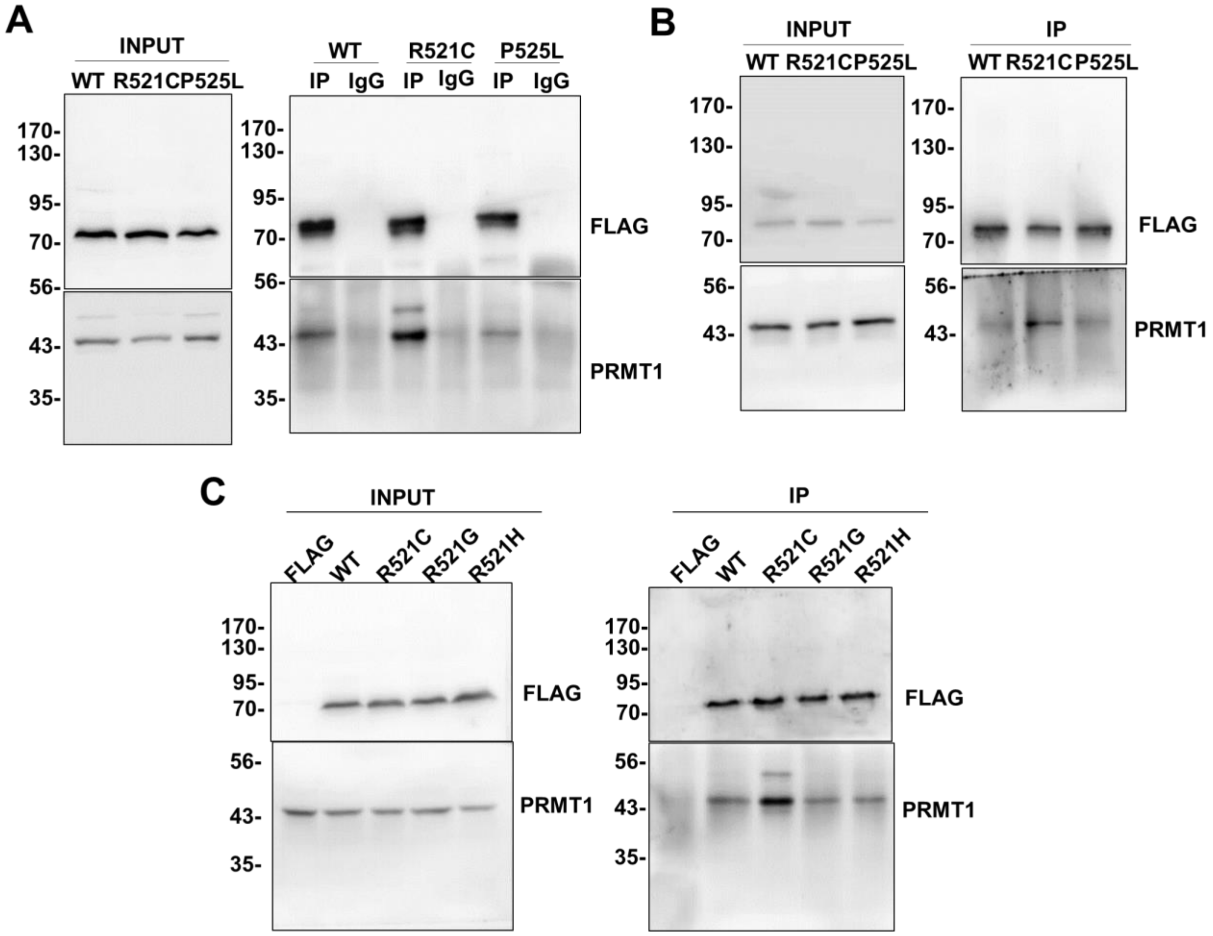

Supplementary Figure 5. PRMT1 was more associated with ALS-associated FUS-R521C than with FUS-WT or FUS-P525L. Full blot images including the data presented in Figure 1B (A), Figure 1D (B), and Figure 1F (C). To analyze FLAG-FUS (70 kDa) and PRMT1 protein (43kDa) on the same blot, the membrane was cut into upper and lower part, and different antibodies (upper: anti-FLAG antibody; lower part: anti-PRMT antibody) were probed.

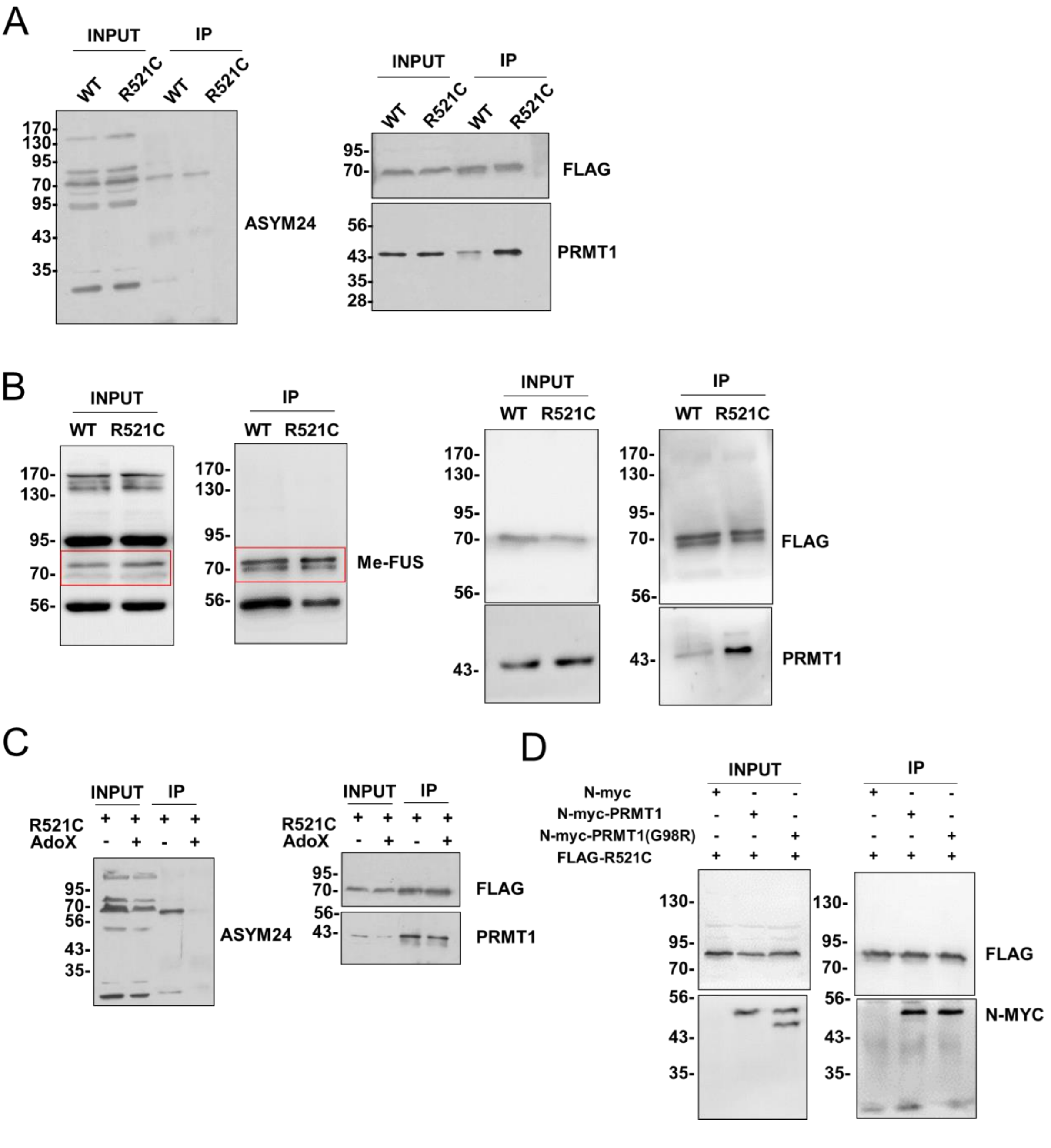

Supplementary Figure 6. PRMT1 was sequestered into methylated FUS-R521C-positive aggregates. Full blot images including the data presented in Figure 2A (A), Figure 2B (B), Figure 2F (C) and Figure 2G (D). Red box in supplementary Figure 7B indicates the crooped gel blot presented in Figure 2B. To analyze FLAG-FUS (70 kDa) and PRMT1 protein (43kDa) on the same blot, the membrane was cut into upper and lower part, and different

antibodies (upper: anti-FLAG antibody; lower part: anti-PRMT antibody or anti-Myc antibody) were probed.

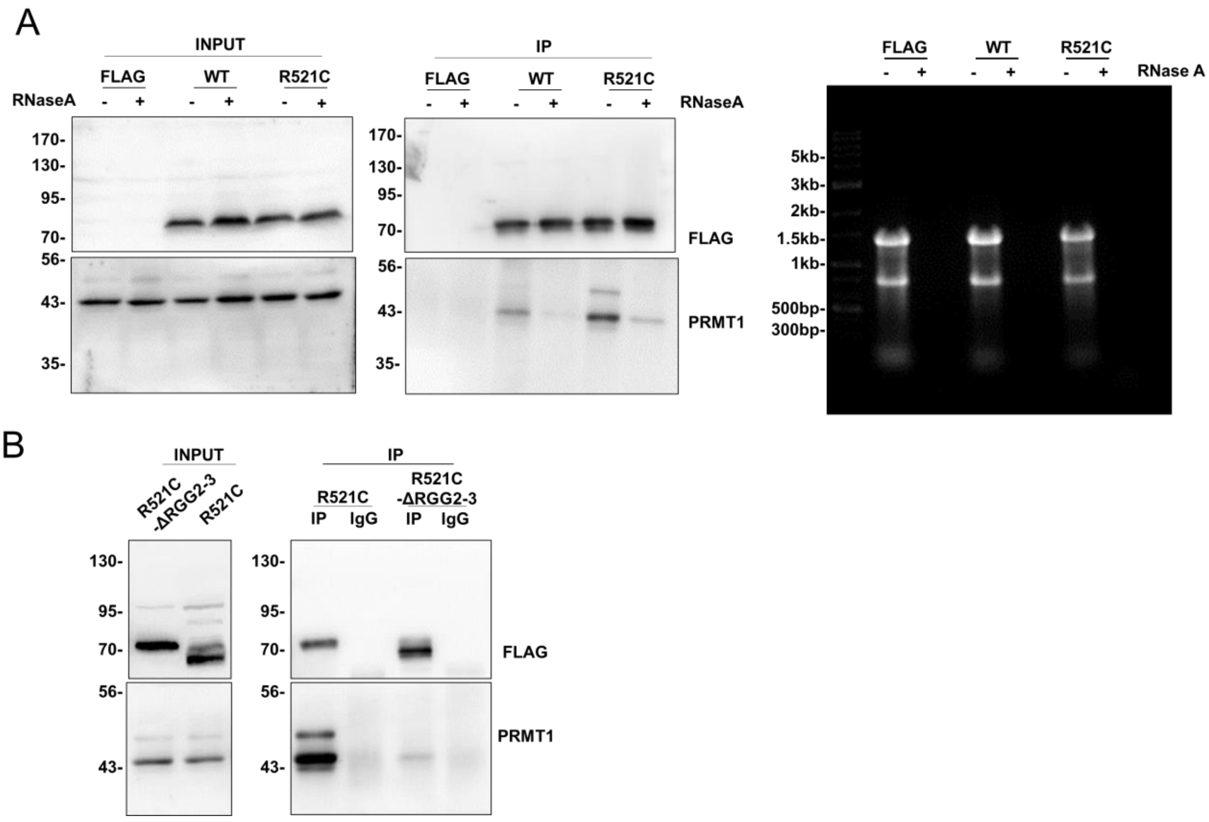

Supplementary Figure 7. Association between FUS and endogenous PRMT1 is dependent on RNAs and RGG domains.

Full blot or gel images including the data presented in Figure 3B (A) and Figure 3D (B). To analyze FLAG-FUS (70 kDa) and PRMT1 protein (43kDa) on the same blot, the membrane was cut into upper and lower part, and different antibodies (upper: anti-FLAG antibody; lower part: anti-PRMT antibody) were probed.

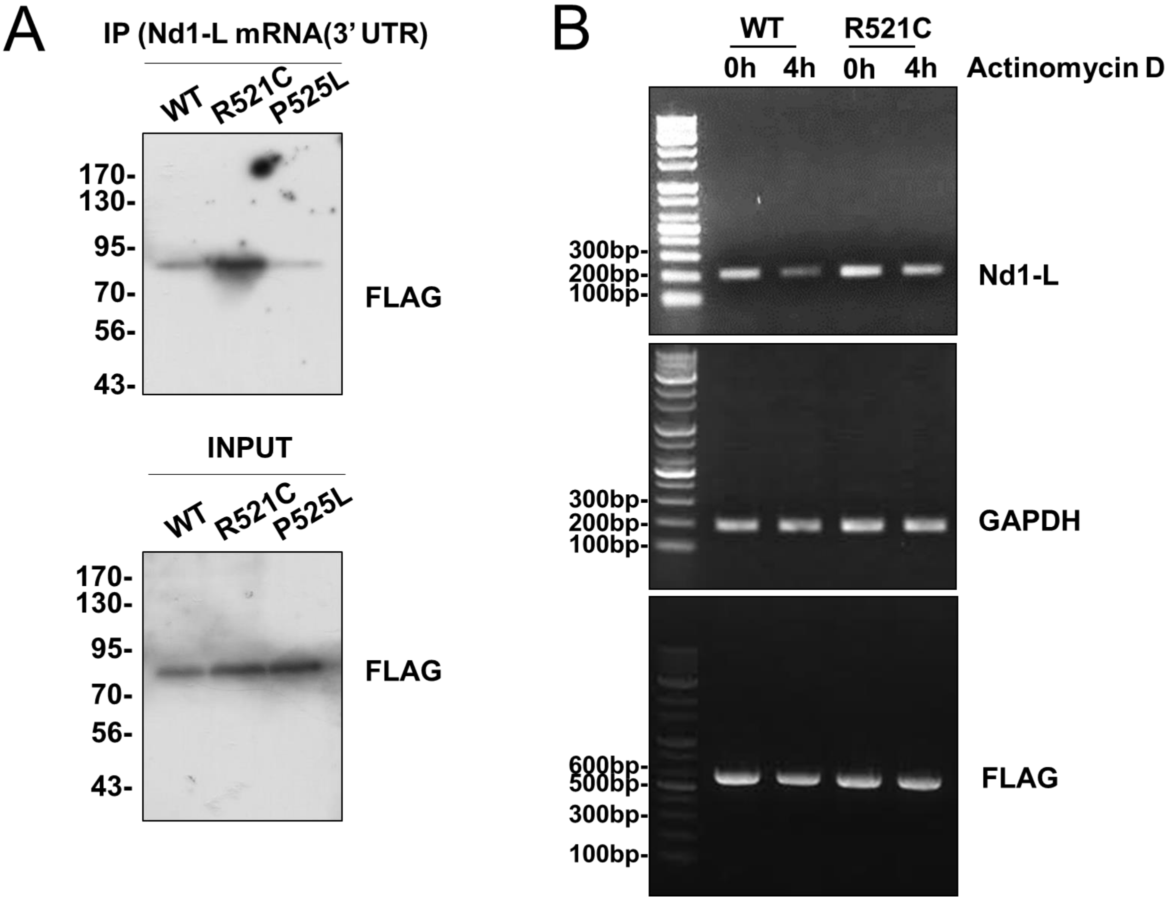

Supplementary Figure 8. ND1-L mRNA is sequestered into FUS-R521C-PRMT1 complexes. Full blot or gel image including the data presented in Figure 6A (A) and Figure 6D (B).

Supplementary Table 1. List of primer for cloning

Name	Direction	Sequence (5'→3')
FLAG-FUSWT	Sense	CGCCCAAGCTTATGGCCTCAAACGATTATAC
	Antisense	CGCGGATCCTTAATACGGCCTCTCCCT
FLAG-FUSR521C	Sense	CGCCCAAGCTTATGGCCTCAAACGATTATAC
	Antisense	CGCGGATCCTTAATACGGCCTCTCCCTGCAATCCT
FLAG-FUSP525L	Sense	CGCCCAAGCTTATGGCCTCAAACGATTATAC
	Antisense	CGCGGATCCTTAATACAGCCTCTCCCTGCGA
FLAG-FUS-R521G	Sense	CGCCCAAGCTTATGGCCTCAAACGATTATAC
	Antisense	CGCGGATCCTAATACGGCCTCTCCCTGCCATCCT
FLAG-FUS-R521H	Sense	CGCCCAAGCTTATGGCCTCAAACGATTATAC
	Antisense	CGCGGATCCTTAATACGGCCTCTCCCTGTGATCCT
FLAG-FUS-ΔRGG-R521C	Sense	TTTGCTACTCGCGACAGAGGTGGCTTTGGC
	Antisense	GCCAAAGCCACCTCTGTCGCGAGTAGCAAATGAGAC
AAV-FLAG-FUSWT	Sense	CCGGTCGACGCCACCATGGACTACAAAGACCAT GAC
	Antisense	CCGCTCGAGTTAATACGGCCTCTCCCTGCGATCCTG
AAV-FLAG-FUSR521C	Sense	TTTGCTACTCGCGACAGAGGTGGCTTTGGC
	Antisense	CCGCTCGAGTTAATACGGCCTCTCCCTGCAATCCTG
PRMT1 shRNA	Human	GACATGACATCCAAAGATT
	mouse	GATTGTCAAAGCCAACAAG
Nd1-L shRNA	mouse	GCAGGTCTGCGGAGATTAT
Human GAPDH(Semi-PCR)	sense	TGCACCACCAACTGCTTA
	Antisense	TAGAGGCAGGGATGATGTTC
Human Nd1-L(Semi-PCR)	sense	GCCCATGTCTCCTATGCAGT
	Antisense	GCCCATGAGTACAGCCATTT-
Mouse PRMT1 shRNA#1	Sense	GAGGAACGACTACGTGCAC

Mouse PRMT1 shRNA#2	Sense	GATTGTCAAAGCCAACAAG
Mouse PRMT1 shRNA#3	Sense	GACAAGGTGGTGCTGGATG
Mouse Nd1-L shRNA#1	Sense	GCAGGTCTGCGGAGATTAT
Mouse Nd1-L shRNA#2	Sense	AGTTGACGCTTATATTCAG
Mouse scrambled shRNA	Sense	AACAGTCGCGTTTGCGACTGG
PRMT1	Sense	ACCGCTCGAGATGGCGGCAGCCGAGGCC
	Antisense	CGCCAAGCTTGCGCATCCGGTAGTCGGT

Supplementary Method

Nuclear and cytosol fractionation and IP

Hela cells expressing 3XFLAG-FUS-WT, orFUS-R521C were lysed with cytoplasmic extraction buffer (10 mM HEPES pH 7.9, 10 mM KCl, 0.1 mM EDTA, 1.5 mM $MgCl_2$, 1 mM DTT, 0.2% Nonidet P-40, and protease inhibitor). Cells firstly were centrifuged at 14,000 rpm at 4℃ for 5min and then the supernatant was collected as cytoplasmic fraction. The pellets were incubated with nuclear extraction buffer (20 mM HEPES pH 7.9, 420 mM NaCl, 0.1 mM EDTA, 1.5 mM $MgCl_2$, 1 mM DTT and protease inhibitor) at room temperature for 5min. Extracted cells with nuclear extraction buffer were centrifuged at 14,000 rpm at 4℃ for 5min. The supernatant was collected as nuclear fraction. For coimmunoprecipitation using anti-FLAG antibodies, lysates fractionated from nucleus or cytosol was incubated with anti-FLAG at 4℃ for overnight and then bounded with Protein G-PLUS agarose beads (Santa Cruz Biotechnology Inc., Dallas, TX, USA) at 4℃ for 7h and further performed Western blotting using anti-FLAG, or anti-PRMT1. Successful nuclear or cytosolic fractionation was confirmed by Western blot analysis using anti-Laminin(A/C) as a nuclear marker or anti-GAPDH as a cytosolic marker.
